# MiR-6875-3p promotes the proliferation, invasion and metastasis of hepatocellular carcinoma via BTG2/FAK/Akt pathway

**DOI:** 10.1186/s13046-018-1020-z

**Published:** 2019-01-08

**Authors:** Yingjun Xie, Jian Du, Zefeng Liu, Dan Zhang, Xiaoxiao Yao, Yongsheng Yang

**Affiliations:** 1 Department of Hepatobiliary Pancreatic Surgery, The Second Hospital of Jilin University, Changchun, 130041 Jilin People’s Republic of China; 2 Department of Hepatobiliary Surgery, The First Affiliated Hospital of Dalian Medical University, Dalian, 116011 Liaoning People’s Republic of China

**Keywords:** miR-6875-3p, BTG2, Proliferation, Invasion, Metastasis, HCC

## Abstract

**Background:**

Increasing evidence supports the association of microRNA with tumor occurrence and development. However, the expression of miR-6875-3p and its role in cell proliferation, invasion and metastasis in hepatocellular carcinoma (HCC) remains elusive.

**Methods:**

The expression of miR-6875-3p and BTG2 in HCC tissues and cell lines was detected by using in situ hybridization, immunohistochemistry and qRT-PCR, respectively. A western blot assay, qRT-PCR and Luciferase reporter assay were employed to study the interaction between miR-6875-3p and BTG2. Cell proliferation invasion and metastasis were measured by MTT, transwell and matrigel analyses in vitro. In vivo, tumorigenicity and metastasis assays were performed in nude mice.

**Results:**

We found that miR-6875-3p were elevated expressed in HCC tissues and cell lines, and negatively correlated with BTG2 expression, while positively correlated with tumor staging, size, degree of differentiation, and vascular invasion of HCC. Moreover, in vitro and in vivo assays showed that miR-6875-3p regulates EMT and improve the proliferation, metastasis and stem cell-like properties of HCC cells. BTG2 was identified as a direct and functional target of miR-6875-3p via the 3’-UTR of BTG2. We also confirmed that miR-6875-3p plays its biological functions via the BTG2/FAK/Akt pathway.

**Conclusion:**

Our study provides evidence that high expression of miR-6875-3p can promote tumorigenesis of HCC in vitro and in vivo, so as to function as a novel oncogene in HCC. In mechanism, we found that miR-6875-3p plays its biological functions via the BTG2/FAK/Akt pathway.

**Electronic supplementary material:**

The online version of this article (10.1186/s13046-018-1020-z) contains supplementary material, which is available to authorized users.

## Introduction

Within worldwide, HCC is the fifth most common malignant tumors and the third cause of cancer-related death currently. Despite advances in HCC treatment in recent years, the survival rate is still poor [1]. The recurrence and metastasis of HCC in the liver is one of the obstacles affecting its therapeutic effect. Therefore, it is urgent to further explore the molecular pathological mechanism underlying cancer progression and metastasis, and it may provide a new therapeutic strategy for improving the prognosis of HCC patients.

MicroRNA (miRNA) is a small endogenous non-coding RNA molecule consisting of approximately 21–25 nucleotides. The miRNA is linked to the 3’-UTR of the target gene, resulting in decreased mRNA expression or transcriptional inhibition of protein [2]. Currently, growing studies have shown that miRNA, as a proto-oncogene or tumor suppressor gene, mediates cell proliferation, apoptosis, invasion and metastasis through various signaling pathways, thus playing a crucial regulation role in tumor development and prognosis [3]. As a member of the miRNA family, miR-6875 has been reported to be highly expressed in early breast cancer patients [4]. Kijima [5] demonstrated that poor tumor immunotherapy effect and prognosis occurred in metastatic colorectal cancer patients with high expression of miR-6875. These studies suggest that miR-6875 may play an important role in the development of tumors. Furthermore, our previous studies have shown that miR-6875-3p expression was significantly increased in HCC tissues compared with para-carcinoma tissues using microarray analysis (*P* < 0.05, Additional file 1: Figure S1). However, its biological functions and correlation with HCC prognosis have not been reported yet and need to be further explored.

BTG2, also known as PC3/APRO1/TIS21, is a member of the BTG/TOB family. Located on chromosome 1q32.1, this gene encodes 158 amino acids [6]. Studies have found that the expression of BTG2 is decreased in various tumor tissues such as prostate cancer, renal cell carcinoma and HCC [7–9], and it has been confirmed to be associated with poor prognosis of breast cancer, bladder cancer and lung cancer [10–12]. There is evidence that BTG2, as a tumor suppressor gene, plays an vital role in regulating the differentiation, proliferation, apoptosis and migration of tumor cells [13]. Using miRNA target prediction algorithms, BTG2 was detected to be one of the potential target genes of miR-6875-3p.

The role of miR-6875-3p in the invasion and metastasis of HCC was analyzed in this experiment. The results indicated that miR-6875-3p expression was abnormally elevated in HCC tissues, and was positively correlated with low expression of BTG2, vascular invasion, poor differentiation, TNM staging and prognosis. In vitro and in vivo experiments showed that miR-6875-3p regulates EMT, enhances stem-like cell characteristics, and promotes the proliferation, invasion and metastasis of HCC cells. We also found that miR-6875-3p played it biological function by modulating the BTG2/FAK/Akt signaling pathway.

## Material and methods

### Cell cultures

The HCC cell lines (HL7702, Huh7, SNU-449, HepG2, HCCLM3, BEL-7404, MHCC97H) were bought from the American Type Culture Collection. All cell lines were cultured in Dulbecco’s modified Eagle’s medium (Gibco, USA) containing 10% foetal bovine serum(Gibco, USA) in a 37 °C, 5% CO_2_ incubator.

### Patient specimens

This study was approved by the Ethics Committees of the Second Affiliated Hospital of Jilin University. A total of 108 HCC samples were obtained from patients with HCC aged from 36 to 77 years, all of whom underwent radical surgical resection at the Second Affiliated Hospital of Jilin University between January 2010 and December 2012. None of these patients underwent chemotherapy or radiotherapy before surgery. Tumorous and adjacent non-cancerous tissues were frozen in liquid nitrogen after the surgical resection for further examination. Tumour stage was classified according to pTNM classification advocated by the International Union against Cancer and were followed up until June 2018. We obtained written informed consent from all of the patients in accordance with the Declaration of Helsinki.

### In situ hybridization (ISH)

ISH analysis was performed as the method described before [14]. Antisense oligonucleotide probes for miR-6875-3p (Exiqon Inc., Woburn, MA, USA) were used for ISH.

### Immunohistochemistry

Immunohistochemical analysis was performed as previously described [14]. Tumorous and adjacent non-cancerous tissue sections were hybridized with diluted primary antibody against BTG2 (1:200, Santa Cruz, USA) at 4 °C overnight. The positive cells were counted under a microscope (Olympus Vanox-T, Hamburg, Germany) and analyzed according to published protocols.

### Western blot

Cells and tissues were washed once with ice-cold phosphate-buffered saline (PBS) containing 100 mM sodium orthovanadate and solubilized in lysis buffer. The supernatant was collected after centrifugation. Then the protein concentration was measured using a BCA Protein Quantitation Assay (Pierce, USA). A total of 20 μg of each protein sample was separated on a 10% gradient polyacrylamide gel and transferred onto polyvinylidene fluoride (PVDF) membranes. The membranes were incubated in a primary antibody against BTG2, E-cadherin,α-catenin, N-cadherin, vimentin, β-actin, p-FAK(Tyr576, Tyr925), FAK, p-Akt, and Akt (Cell Signaling, USA), overnight at 4 °C. After incubation in a secondary antibody for 2 h, the targeted proteins in the membrane were detected using an enhanced chemiluminescence system. The intensities of the bands were qualified by Image J (National Institutes of Health, Bethesda, MD, USA).

### Quantitative real-time RT-PCR

Total RNA from the cells or tissues was isolated with TRIzol Reagent (Invitrogen) and reverse transcribed to cDNA with ExScript RT Reagent (Takara) according to the manufacturer’s protocol. Real-time RT-PCR was carried out using Platinum SYBR Green qPCR SuperMix-UDG reagents(Invitrogen) on the PRISM 7900HT system (Applied Biosystems). β-actin expression was used to normalize for variance. The expression levels of specific genes are reported as ratios of expression of β-actin in the same master reaction.

### miRNA and shRNA transfection

The shRNA targeting the BTG2, miR-6875-3p mimic, inhibitor and the negative control were obtained from Ribobio (Guangzhou, China). Cells (5 × 105 cells/2 ml/well) were plated at 60% confluence in a six-well plate in DMEM without antibiotics. After 48 h, miR-6875-3p mimic, inhibitor or negative control oligonucleotide was transfected into cells with Lipofectamine 2000 at a final concentration of 50 nM according to the instructions of the manufacturer’s. After 4–6 h, the medium was replaced with fresh DMEM containing 10% FCS and the cells were cultured for further experiment. The transfection of shRNA was performed as described above. To inhibit FAK phosphorylation, dissociated cells were incubated with PF573228 (10 μM, Sigma-Aldrich, Saint Louis, MO, USA) for 30 min at 37 °C before Western blot analysis.

### Luciferase reporter assay

The 3’-UTR of BTG2 containing the predicted binding site of miR-6875-3p was amplified from normal fetal genomic DNA using PCR specific primers. The PCR product was restricted and inserted between the restriction sites SpeI and HindIII into pMIR-REPORT-basic vector (Applied Biosystems, USA). The consensus miR-6875-3p binding site was mutated via PCR using a QuikChange II XL site-directed mutagenesis kit (Stratagene, USA). All clones were verified by DNA sequencing.Then, miR-6875-3p mimic or negative control was co-transfected with luciferase reporter vectors into HCC cells as described above, followed by the detection of luciferase activity via a luminescence reporter gene assay system (PerkinElmer, Norwalk, CT, USA) according to the manufacturer’s instructions.

### MTT assay

3-(4, 5-dimethylthiazol-2-yl)-2, 5-diphenyltetrazolium bromide (MTT) solution was used to detect the proliferative rate of indicated cell lines. MTT were added (final concentration 0.5 mg/ml, stock solution 5 mg/ml MTT in phosphate-buffered saline) in every group cells for 3 h. Cells were lysed in acidified 2-propanol and absorbance measured at 490 nm.

### Animal experiment

The research was conducted in accordance with the declaration of Helsinki and with the guide for care and use of laboratory animals as adopted and promulgated by the United National Institutes of Health. Animal handling and research protocols were approved by the Animal Care and Use Ethnic Committee. Male BALB/c nude mice aged 4 to 6 weeks were purchased form Shanghai Slac Laboratory Animal Co. Ltd. (Shanghai, China). Cells were subcutaneously injected into the flank region of the mice. The animals were sacrificed 5 weeks post inoculation. Tumors were surgically removed and weighed. The tumor metastatic ability was determined by tail vein injection of the cells into male nude mice. After 6 weeks, the animals were ether-anesthetized, and their lungs were removed to determine the pulmonary metastatic foci.

### Transwell and matrigel assay

The cell migration and invasion were assessed using the wound-healing and transwell assays as previously described [15].

### Magnetic cell sorting

CD31 and EPCAM positive cells were isolated from HL7702 and Huh7 cells using anti-CD31 and anti-EPCAM antibodies coupled to magnetic beads (Miltenyi Biotech) using the MACS system (Miltenyi Biotech).

### Colony formation assays

The cells were plated in a six-well plate at 5 × 10^2^ per well and incubated for 2 weeks for the colony formation assay. The cells were washed twice with PBS, fixed with methanol/acetic acid (3:1, *v*/v), and stained with 0.5% crystal violet (Sigma, China). The number of colonies was counted under a microscope (Olympus Vanox-T, Hamburg, Germany).

### Statistical analysis

Data were recorded as the means ± standard deviation (SD). Survival analysis was analyzed using Kaplan-Meier method. Association between miR-6875-3p and BTG2 expression in HCC tissues was calculated using Spearman rank correlation test. The χ^2^ test was performed to analyze the relationship between miR-6875-3p expression and the clinicopathological characteristics. The differences between the groups were undertaken using the Student two-tailed t test. A *p* < 0.05 was considered statistically significant. The statistical analysis and figure generation were performed using SPSS version 23.0 (IBM).

## Results

### Expression of miR-6875-3p in HCC and its correlation with clinicopathological characteristics

**Fig. 1:**
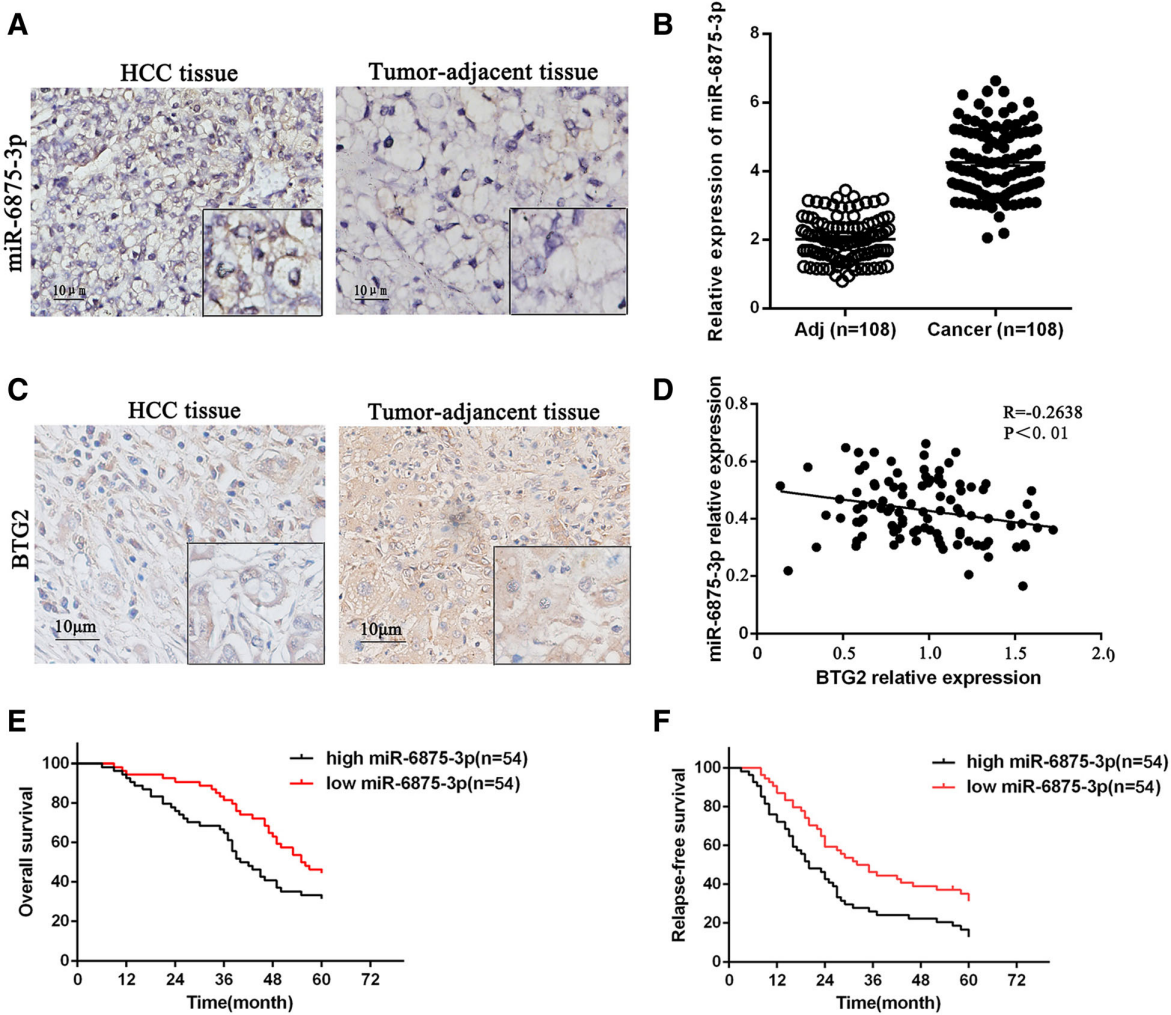
miR-6875-3p expression was inversely associated with BTG2 expression in HCC tissues, and high miR-6875-3p expression predicted Shorter RFS and OS. **a** Analysis of miR-6875-3p expression in HCC tissues and tumor-adjacent tissues using ISH. **b** The expression levels of miR-6875-3p were detected by qRT-PCR in 108 paired HCC and tumor-adjacent tissues. **c** Analysis of BTG2 expression in HCC tissues and tumor-adjacent tissues via IHC. **d** Expression of miR-6875-3p was inversely correlated with the mRNA level of the BTG2 (*R* = − 0.2638, *p* < 0.01, Spearman’s correlation analysis). **e, f** Kaplan-Meier analysis suggested that the OS and RFS rates of the low miR-6875-3p expression group were higher than those of the high miR-6875-3p expression group (*p* < 0.05). Scale bars, 50 mm. Data are shown as the mean ± SD of three replicates (*p < 0.05)

In order to investigate the expression of miR-6875-3p in HCC, miR-6875-3p was detected in 108 HCC and paracancerous tissue specimens through ISH and qRT-PCR. The ISH results showed that miR-6875-3p expression was significantly increased in HCC tissues compared with paracancerous tissues (*P* < 0.05, Fig. 1a). The qRT-PCR analysis also confirmed the above expression pattern (Fig. 1b), indicating abnormally elevated expression of miR-6875-3p in HCC tissues. Furthermore, we detected the BTG2 expression in 108 specimens using IHC. It was shown that the expression of BTG2 in HCC tissues was significantly lower than that in paracancerous tissues (*P* < 0.05, Fig. 1c), and was negatively correlated with the expression level of miR-6875-3p (Fig. 1d).

To further investigate the clinicopathological significance of miR-6875-3p, we analyzed the correlation between miR-6875-3p expression and clinicopathological characteristics in 108 HCC patients. Patients were divided into two groups according to the median expression level of miR-6875-3p. As shown in Table 1, the miR-6875-3p expression is correlated with the TNM staging (*P* = 0.001), tumor size (*P* = 0.001), degree of differentiation (*P* = 0.017) and vascular invasion (*P* = 0.009) of HCC. Meanwhile, these patients were followed up for 5 years. Kaplan-Meier analysis confirmed significantly decreased OSR and DFS of patients in miR-6875-3p high-expression group compared with those in the low-expression group (*P* < 0.05, Fig. 1e, f). These results revealed a correlation between high expression of miR-6875-3p and poor prognosis of HCC.

**Table 1:** Relationship between miRNA-6875-3p and clinicopathological parameters in 108 HCC patients

Variables	All cases	miR-6875 expression		*P* ^*^
		High *N* = 54	Low *N* = 54	
*Age(years)*				
>50	61	32	29	
≤ 50	47	24	23	0.885
*Gender*				
Male	72	39	33	
Female	36	20	16	0.890
*Tumor number*				
Single	87	41	46	
Multiple	21	13	8	0.224
*Etiology*				
viral	93	46	47	
Non-viral	15	7	8	0.841
*Serum AFP (ng/ml)*				
>200	48	22	26	
≤ 200	60	33	27	0.344
*Tumor stage*				
I/II	63	22	41	
III/IV	45	33	12	0.001
*Tumor size (cm)*				
>5	58	37	21	
≤ 5	50	16	34	0.001
*Tumor differentiation*				
Well	42	16	26	
Moderate	36	17	19	
Poor	30	20	10	0.017
*Vascular invasion*				
Yes	38	23	15	
No	70	24	46	0.009

^*^P probability, from*χ*^2^ test

### In HCC cells, miR-6875-3p down-regulates BTG2 expression through directly acting on its 3’-UTR

**Fig. 2:**
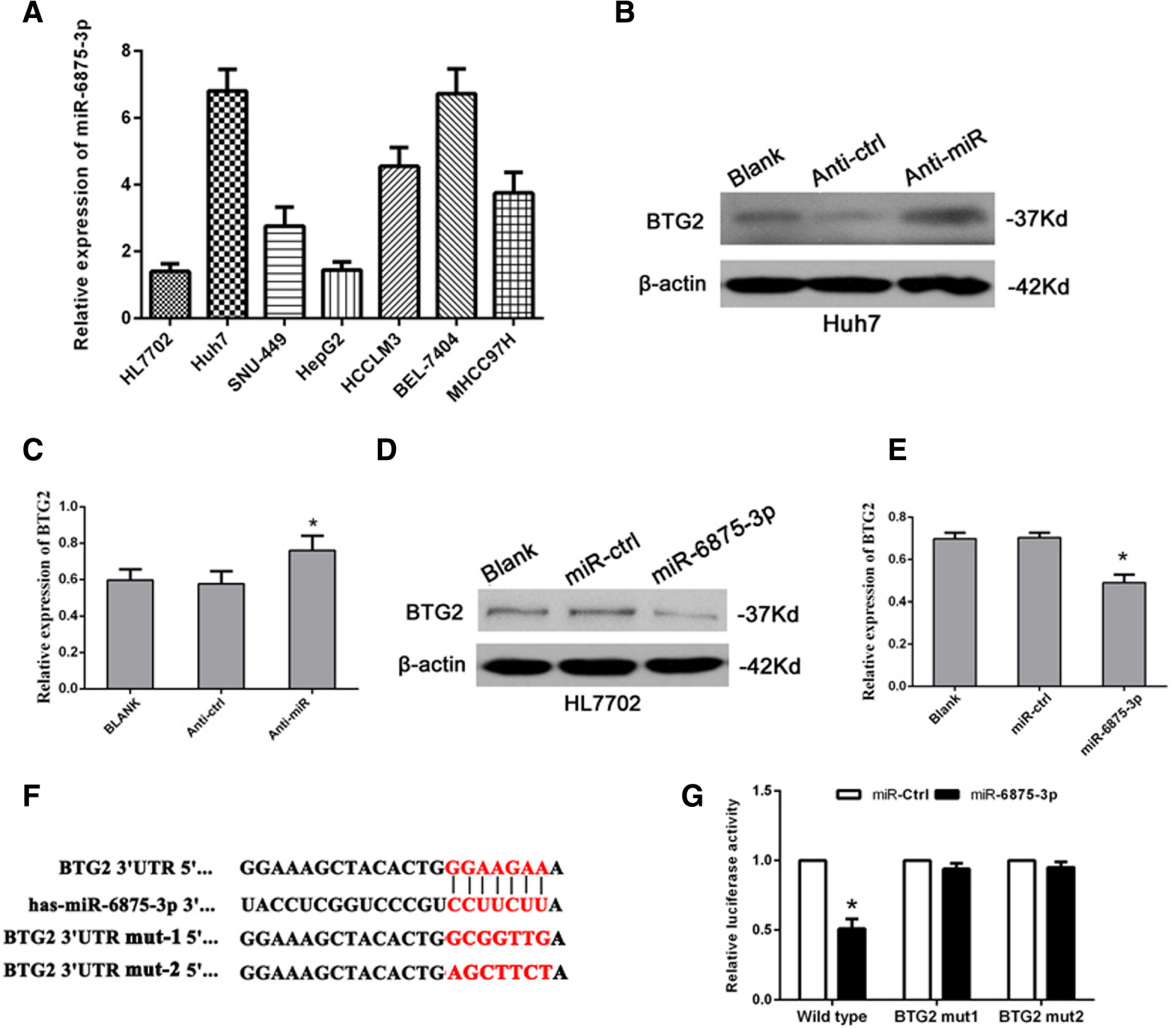
miR-6875-3p downregulated BTG2 expression via directly targeting its 3’-UTR. **a** Expression of miR-6875-3p were examined by qRT-PCR in seven cell lines. **b, c** The protein and mRNA levels of the BTG2 were analyzed after transfection with the miR-6875-3p inhibitor by Western blot and qRT-PCR. **d, e** The protein and mRNA level of the BTG2 were analyzed after transfection with the miR-6875-3p mimic by Western blot and qRT-PCR. **f** The predicted sites of miR-6875-3p binding to the 3’-UTR of the BTG2 were detected via bioinformatics prediction tools. The mutated site in the 3’-UTR of the BTG2 is shown. **g** The effect of miR-6875-3p on luciferase activity induced by the pMIR-BTG2-wt, pMIR-BTG2-mut-1, and pMIR-BTG2-mut-2 reporter plasmids in HL7702 cells was detected via luciferase reporter gene assays. Data are shown as the mean ± SD of three replicates (*p < 0.05)

It was analyzed through miRNA target prediction algorithms (TargetScan, PicTar and miRanda) that BTG2 may be one of the potential target genes of miR-6875-3p. Then, we detected the miR-6875-3p expression in seven HCC cell lines via qRT-PCR. As shown in Fig. 2a, miR-6875-3p was expressed in different degrees in seven cell lines. Of these cell lines, HL7702 and HepG2 had the lower miR-6875-3p expression and Huh7 and BEL-7404 had the higher expression. So we used these four cell lines to perform the following experiments. We transfected miR-6875-3p inhibitor (designated as Anti-miR) and mimic (designated as miR-6875-3p) into Huh7 and HL7702 cells respectively. As shown in the results, compared with the control group (designated as Anti-ctrl and miR-ctrl), BTG2 expression was significantly increased with the knockdown of miR-6875-3p (*P* < 0.05, Fig. 2b, c), but the expression was clearly reduced with the up-regulation of miR-6875-3p (*P* < 0.05, Fig. 2d, e). These results indicated that miR-6875-3p specificity down-regulates BTG2 expression.

To further understand the mechanism by which miR-6875-3p inhibits BTG2, we found that the junction site of miR-6875-3p was located at the 3’UTR of BTG2 (Fig. 2f). Then the target region sequence (wild-type) or mutated sequence 1 (BTG2 mut-1) or 2 (BTG2 mut-2) was cloned into luciferase reporter vector respectively. These vectors were then co-transfected into HL7702 cells together with miR-6875-3p mimic or miR-ctrl. The results showed that overexpression of miR-6875-3p significantly reduced the activity of wild-type luciferase, which was not the case for mutants. (*P* < 0.05, Fig. 2g). These results suggested that miR-6875-3p-mediated regulation of BTG2 expression is achieved by acting on the specific region of BTG2 3’-UTR.

### miR-6875-3p promotes the proliferation of HCC cell and tumorigenicity in vivo

**Fig. 3:**
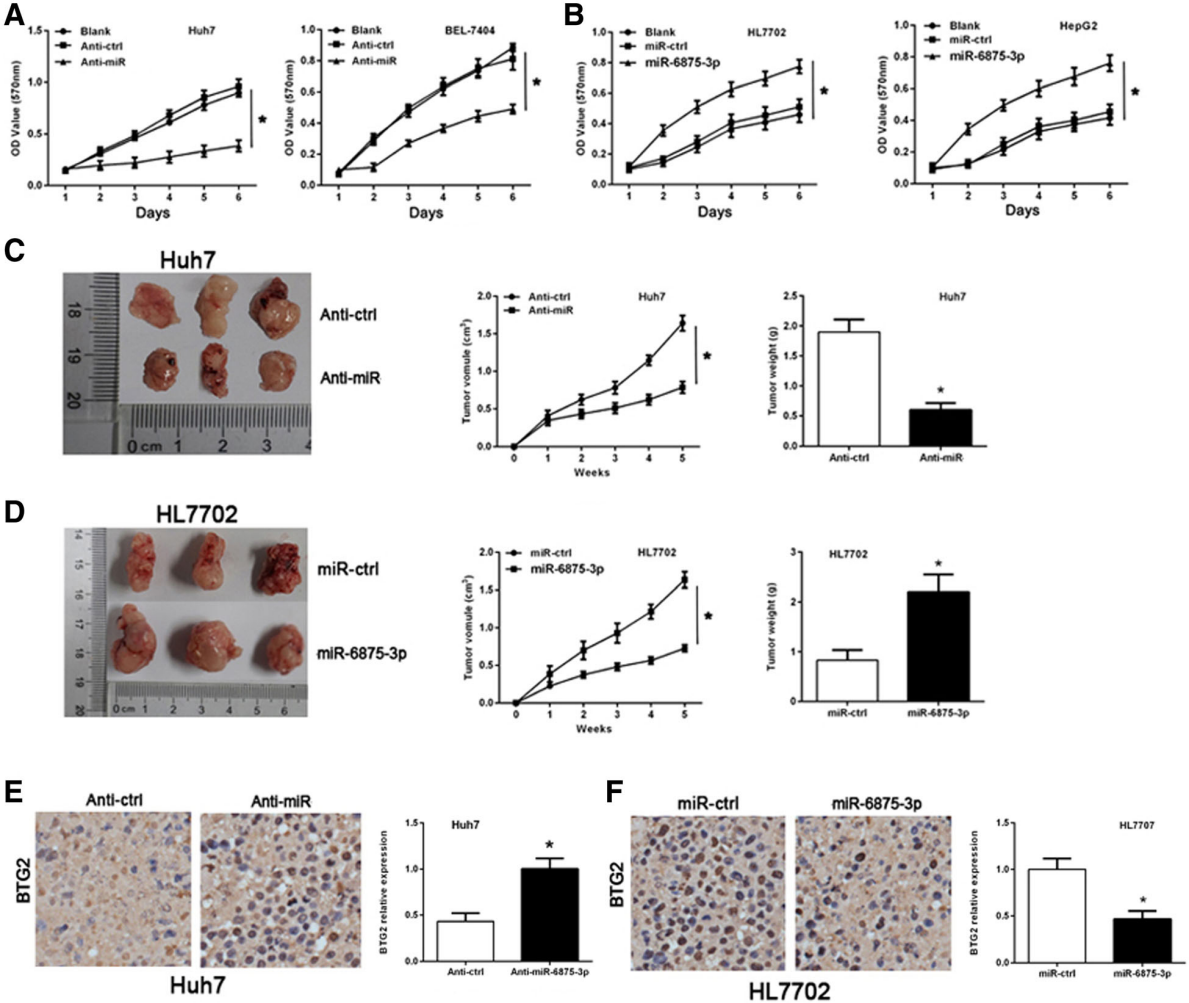
The elevated expression of miR-6875-3p promoted the proliferation of HCC cells and tumorigenicity in vivo. **a, b** miR-6875-3p was down-regulated in Huh7 and BEL7404 cells through the transfection of a miR-6875-3p inhibitor. miR-6875-3p was up-regulated in HL7702 and HepG2 cells via the transfection of a miR-6875-3p mimic. MTT assay was used to analyze the effect of miR-6875-3p on the proliferation of these four cell lines. **c, d** Huh7 cells stably low-expressing miR-6875-3p or empty vector were injected into the flanks of nude mice. HL7702 cells stably overexpressing miR-6875-3p or empty vector were injected into the flanks of nude mice. Surgically removed tumor tissues from nude mice 5 weeks post-inoculation. Tumor volume at different time points and Tumor weight were measured. **e, f** Analysis of BTG2 expression in resected tumors tissues by IHC and Western blot. Data are shown as the mean ± SD of three replicates (*p < 0.05)

In order to investigate the regulation of miR-6875-3p on HCC cell proliferation and tumorigenicity, miR-6875-3p inhibitor was transfected into Huh7 and BEL-7404 cells, and miR-6875-3p mimic was transfected into HL7702 and HepG2 cells to detect the proliferation of cells in each group through MTT assay. The results showed that down-regulation of miR-6875-3p resulted in significant growth inhibition in HCC cells, while overexpression of miR-6875-3p promoted proliferation (*P* < 0.05, Fig. 3a, b). We further inoculated Huh7 cells with low-expression of miR-6875-3p and HL7702 cells with overexpression subcutaneously in the lateral abdomen of nude mice. The mice were killed and the tumors were collected after 5 weeks. The results showed that mice injected with low-expressing miR-6875-3p Huh7 cells (Anti-miR group) exhibited significantly decreased volume and weight of transplanted tumors compared to the control group (*P* < 0.05, Fig. 3c). In contrast, mice injected with miR-6875-3p over-expressing HL7702 cells (miR-6875-3p group) showed significantly larger tumor volume and weight (*P* < 0.05, Fig. 3d). Immunohistochemical analysis demonstrated that the BTG2 expression was significant higher in Anti-miR group, and decreased in miR-6875-3p group (P < 0.05, Fig. 3e, f). These results indicated that miR-6875-3p can promote the HCC cell proliferation and tumor formation in vivo, and further verify that miR-6875-3p negatively regulates the expression of BTG2.

### miR-6875-3p regulates EMT, and enhances the migration, invasion and metastasis of HCC cells

**Fig. 4:**
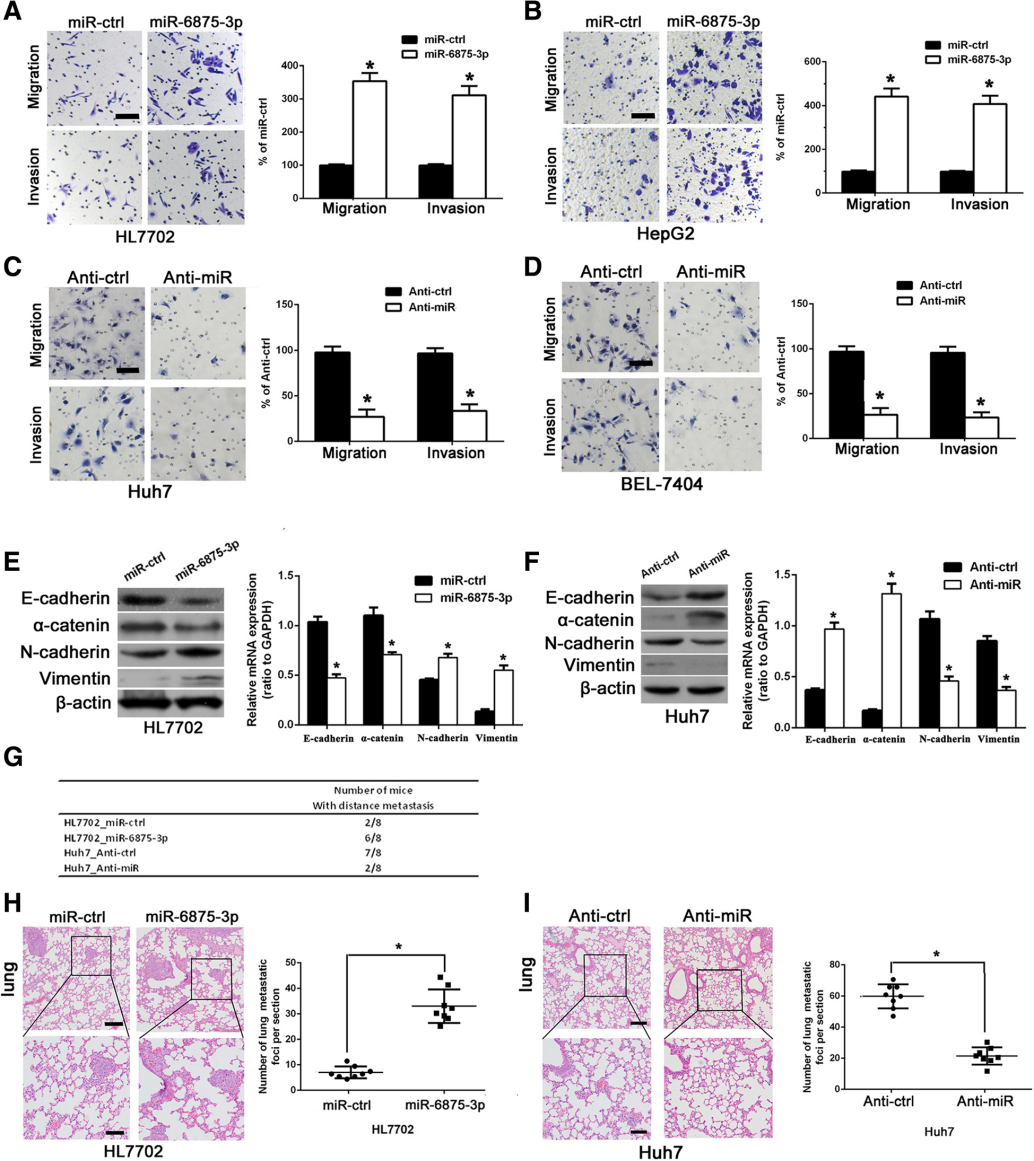
miR-6875-3p regulates the transition between epithelial and mesenchymal phenotypes, and promotes migratory, invasive, and metastatic of HCC cells. **a, b** HL7702 and HepG2 cells via the transfection of a miR-6875-3p mimic were subjected to Transwell migration (top) and Matrigel invasion assays (bottom). Quantification of migrated cells through the membrane and invaded cells through either the membrane or Matrigel for each cell line is shown as a proportion of the vector controls. **c, d** Huh7 and BEL7404 cells via the transfection of a miR-6875-3p inhibitor were subjected to Transwell migration (top) and Matrigel invasion assays (bottom). Quantification of migrated cells through either the membrane or Matrigel for each cell lines is shown as proportions of their vector controls. **e, f** Expression of epithelial (E-cadherin and a-catenin) and mesenchymal (N-cadherin and Vimentin) markers were analyzed by Western blot in transfected HL7702 and Huh7 cells. **g** The total number of mice with distant metastasis at 6 weeks after injection of transfected HL7702 and Huh7 cells. **h, i** The number of metastatic foci per section in lungs from individual mouse with injection of transfected HL7702 and Huh7 cells. Scale bars, 200 nm (**a, b, c** and **d**) and 50 mm (**h** and **i**). Data are shown as the mean ± SD of three replicates (*p < 0.05)

We further evaluated the effect of miR-6875-3p on tumor cell migration and invasion via transwell migration and matrigel assay. Up-regulation of miR-6875-3p significantly increased the migration and invasion capacity of HL7702 and HepG2 cells (*P* < 0.05, Fig. 4a, b). However, silencing miR-6875-3p dramatically inhibited the migration and invasiveness of Huh7 and BEL-7404 cells (*P* < 0.05, Fig. 4c, d). These results indicated that miR-6875-3p promotes the migration and invasion behavior of HCC cells. Furthermore, we detected the relationship of miR-6875-3p and EMT markers expression in HCC tissues using qRT-PCR. The results showed that the expression of miR-6875-3p was negatively correlated with the epithelial markers (E-cadherin and а-catenin), and was correlated with the expression level of mesenchymal markers (N-cadherin and Vimentin) (*P* < 0.05, Additional file 2: Figure S2). Moreover, we revealed that over-expressing miR-6875-3p reduced the levels of epithelial markers (E-cadherin and а-catenin), while increasing the mesenchymal markers (N-cadherin and Vimentin) levels in HL7702 cells (*P* < 0.05, Fig. 4e). Conversely, silencing miR-6875-3p expression reverted to an epithelial phenotype compared with its control group in Huh7 cells (*P* < 0.05, Fig. 4f). These results suggested that overexpression of miR-6875-3p can regulate EMT of HCC cells.

We further injected HL7702 transfected with miR-6875-3p mimic and HuH7 cells transfected with inhibitor respectively into the nude mice through the tail vein to observe their tumorigenic ability in the lungs. We observed that overexpression of miR-6875-3p not only increased the number of mice with tumor formation in the lung, but also significantly increased the number of lung tumors of each mouse (*P* < 0.05, Fig. 4g, h). While silencing miR-6875-3p in Huh7 cells reduced both the number of tumor-bearing mice and lung tumors of each mouse (*P* < 0.05, Fig. 4g, i). Therefore, the in vivo results further indicated that miR-6875-3p plays a crucial role in regulating the tumorigenic ability of HCC cells.

### Down-regulation of BTG2 expression restored the proliferation, migration and invasion of HCC cells

**Fig. 5:**
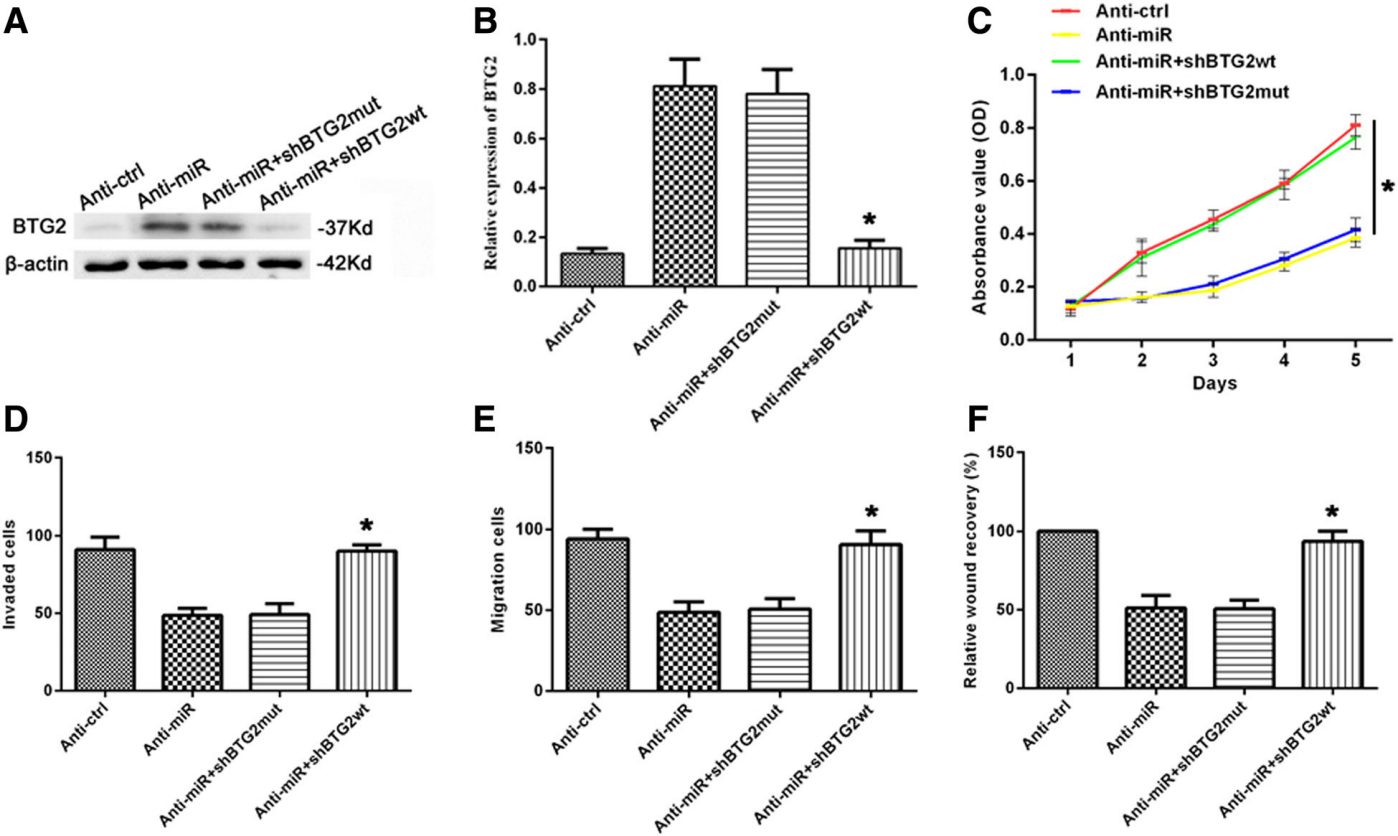
Silencing of BTG2 expression reversed proliferation, invasion and migration of HCC cells. **a, b** Protein and mRNA levels of the BTG2 decreased after transfection with BTG2 shRNA into miR-6875-3p-silenced Huh7 cells, as demonstrated by Western blot and qRT-PCR. **c** Proliferation of Huh7 cells was reversed after transfection with BTG2 shRNA into miR-6875-3p-silenced Huh7 cells, as demonstrated by MTT assay. **d, e** and **f** Transwell and Wound-healing assay demonstrated that Silencing of BTG2 expression ameliorated down-regulated miR-6875-3p-induced suppression of Huh7 cell invasion and migration. Data are shown as the mean ± SD of three replicates (*p < 0.05)

Previous studies have reported that BTG2 can inhibit the proliferation, invasion and metastasis of tumor cells [13]. We hypothesized that silencing BTG2 could reverse the effect of low expression of miR-6875-3p on HCC. To demonstrate this, we co-transfected BTG2 shRNA (shBTG2) with Anti-miR into Huh7 cells, and detected the BTG2 expression in each group through Western blot and qRT-PCR (*P* < 0.05, Fig. 5a, b). MTT, transwell and wound-healing assays showed that silencing BTG2 significantly reversed the inhibitory effect of Anti-miR on proliferation, migration and invasion of Huh7 cells, respectively (*P* < 0.05, Fig. 5c-f). The results indicated that BTG2, as a downstream target gene of miR-6875-3p, has an important role in the proliferation, migration and invasion of HCC cells.

### miR-6875-3p promotes the proliferation, migration and invasion of HCC via the BTG2/FAK/Akt pathway

**Fig. 6:**
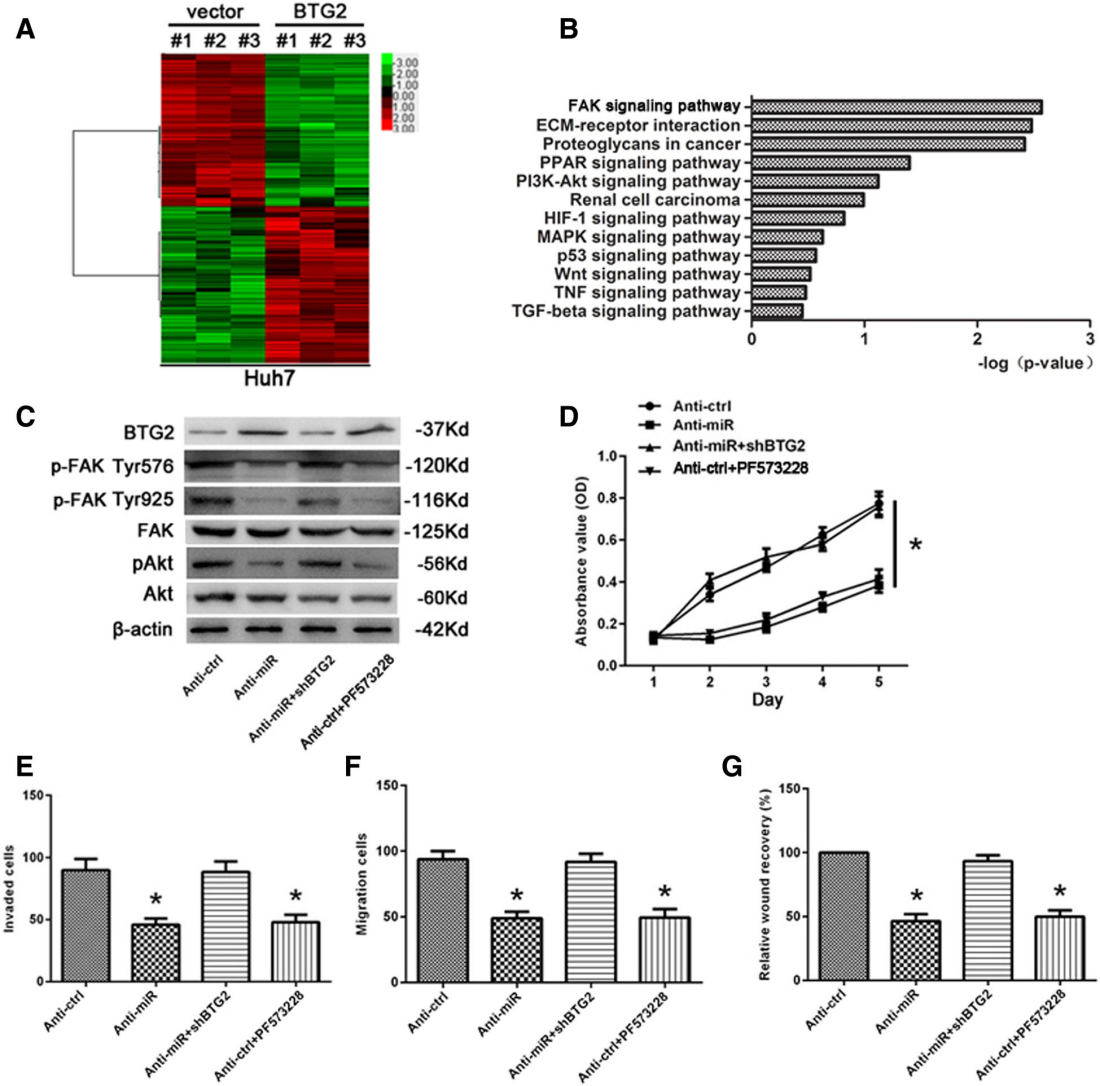
miR-6875-3p-BTG2 induced promotion of proliferation and migration through FAK/Akt pathway. **a** Supervised hierarchical clustering of the genes differentially expressed after BTG2 overexpression in Huh7 cells. **b** Gene-set enrichment analysis was carried out using ConceptGen. **c** Levels of the BTG2, phosphorylated FAK and phosphorylated Akt were detected using Western blot analysis. miR-6875-3p-silenced Huh7 cells were treated with shBTG2 RNA and Huh7 cells were treated with 10 μM of PF573228. **d** The proliferation-promoting effect of miR-6875-3p on Huh7 cells was blocked by PF573228, as demonstrated by an MTT assay. **e, f** and **g** The invasion and migration-promoting effect of miR-6875-3p on Huh7 cells was blocked by PF573228, as demonstrated by transwell and wound-healing assay. Data are shown as the mean ± SD of three replicates (*p < 0.05)

To further study the mechanism of miR-6875-3p-BTG2 axis regulating HCC proliferation and metastasis, we transfected BTG2 gene into Huh7 cells and detected the change of gene expression in cells using gene expression profiling. Microarray analysis showed that numbers of genes significantly differentially expressed afrer regulating BTG2 expression (Fig. 6a), and the FAK pathway was the primary related signaling pathway (Fig. 6b).

Previous studies have shown that activation of FAK/Akt pathway is closely related to the invasion and metastasis of HCC [16]. Therefore, we concluded that miR-6875-3p-BTG2 promoted the proliferation and metastasis via the FAK/Akt signaling pathway. Western blot results showed that the phosphorylation levels of FAK and Akt were downregulated by miR-6875-3p low-expression, while silencing BTG2 eliminated the inhibition of low-expressed miR-6875-3p on FAK/Akt phosphorylation level. When Huh7 cells were treated with PF573228 (FAK phosphorylation inhibitor), the phosphorylation level of Akt also decreased, while the BTG2 expression was increased, which might be induced by negative feedback (Fig. 6c). These results suggested that miR-6875-3p inhibits BTG2 expression, thus activating the FAK/Akt pathway. We further detected functional changes of Huh7 cells in each group via MTT, transwell and wound-healing assays. As shown in the results, silencing BTG2 significantly reversed the inhibitory effect of low-expressed miR-6875-3p on the proliferation, migration and invasion. The above functions of Huh7 cells treated with PF573228 were also significantly inhibited (*P* < 0.05, Fig. 6d-g). These results indicated that miR-6875-3p plays its biological functions via the BTG2/FAK/Akt pathway.

### miR-6875-3p promotes the occurrence of stem cell-like biological behavior in HCC cell lines

**Fig. 7:**
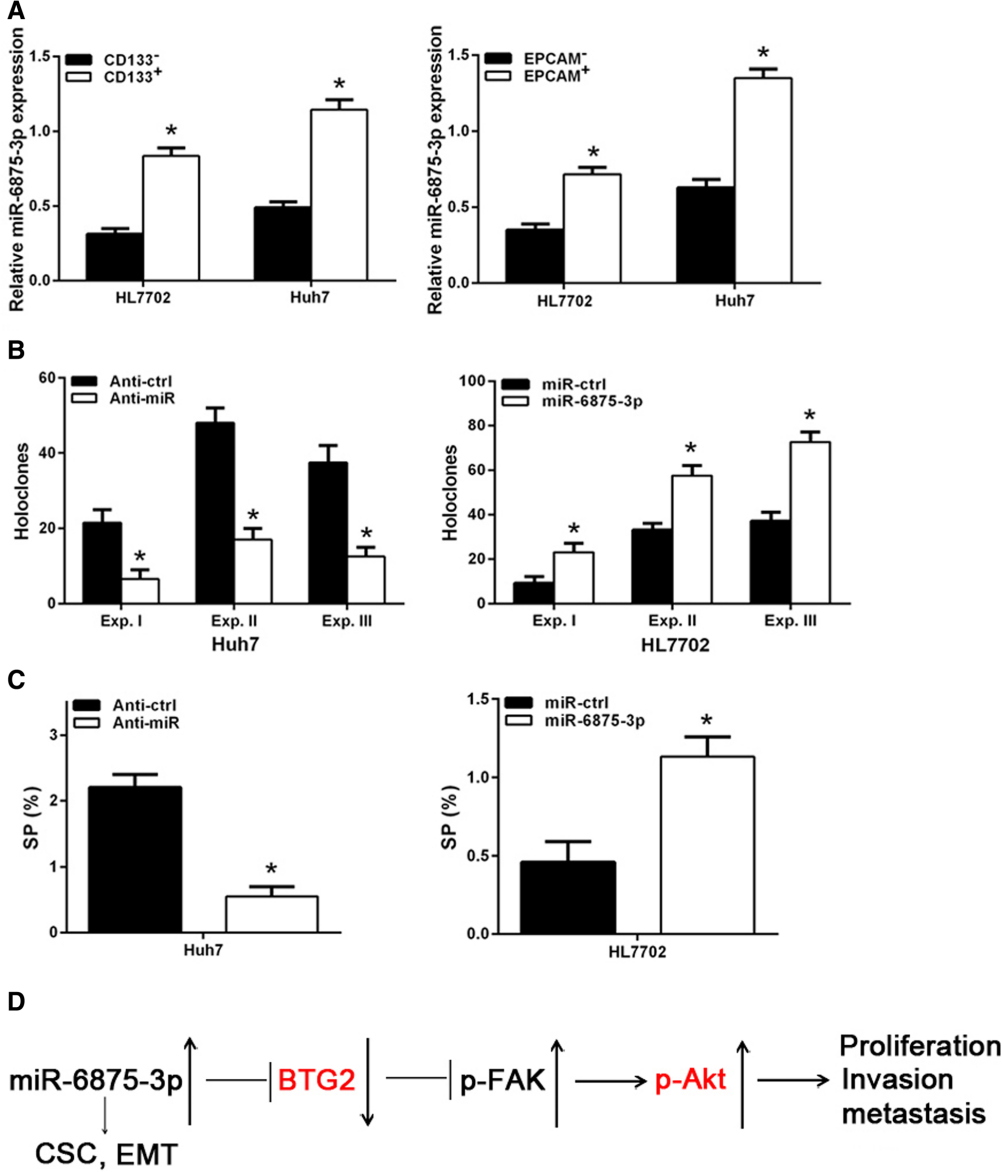
miR-6875-3p promoted emergence of stem cell-like behavior in hepatocellular carcinoma cells. **a** Levels of miR-6875-3p in magnetically sorted CD133^+^ and CD133^−^ were measured by qRT-PCR in HL7702 and Huh7 cells (left). Levels of miR-6875-3p in magnetically sorted EPCAM^+^ and EPCAM^−^ were measured by qRT-PCR in HL7702 and Huh7 cells (right). **b** Holoclone assays in Huh7 cells transfected with miR-6875-3p inhibitor or empty vector (left) and HL7702 cells transfected with miR-6875-3p mimic or empty vector (right) were used in three experiments (Exp. I, 100 cells/well scored on day 9; Exp. II, 100 cells/well scored on day 13; and Exp. III, 500 cells/well scored on day 7). **c** SP population in miR-6875-3p-overexpressing and silenced cells was determined by Hoechst 33342 efflux assays. **d** miR-6875-3p promotes the proliferation, invasion and metastasis of HCC cells via the BTG/FAK/Akt pathway. Data are shown as the mean ± SD of three replicates (*p < 0.05)

There is increasing evidence that EMT is associated with the functional properties of stem cells in tumor cells [17]. Therefore, in order to determine whether miR-6875-3p can regulate certain stem cell-related properties, we first selected CD133^+^ or EPCAM^+^ HL7702 and Huh7 cells, and the qRT-PCR results showed that miR-6875-3p in the positive group was abnormally highly expressed compared to the CD133^−^ or EPCAM^−^ group (P < 0.05, Fig. 7a). Moreover, down-regulation of miR-6875-3p expression inhibited holoclone formation in Huh7 cells; whereas overexpression of miR-6875-3p promoted this ability of HL7702 cells. Previous studies have shown that SP (side population) cells have the characteristics of cancer stem cells (CSC) and can enrich the number of CSC [15]. Similarly, this experiment showed that down-regulated miR-6875-3p in Huh7 cells significantly reduced the percentage of SP cells; whereas up-regulation of miR-6875-3p in HL7702 cells significantly increased the abundance of SP cells (*P* < 0.05, Fig. 7b, c). These results show that miR-6875-3p enhances emergence of stem cell-like behavior in HCC cell lines.

## Discussion

Increasing evidences have demonstrated that miRNAs play an important role in the occurrence and development of tumors. In this study, miR-6875-3p was found to be abnormally highly expressed in HCC tissues and cell lines, and negatively correlated with BTG2 expression while positively correlated with tumor staging, size, degree of differentiation, and vascular invasion of HCC. Moreover, in vitro and in vivo experiments have shown that miR-6875-3p regulates EMT and improve the proliferation, metastasis and stem cell-like properties of HCC cells. BTG2 was first identified as a direct and functional target of miR-6875-3p via the 3’-UTR of BTG2. We also confirmed that miR-6875-3p plays its biological functions via the BTG/FAK/Akt pathway (Fig. 7d).

So far, the study on the correlation between miR-6875-3p and tumors is limited. Metastatic colorectal cancer patients with high expression of miR-6875 and miR-6826 have been reported to have poor immunotherapy effect and poor prognosis [5]. miR-6875 can be used in combination with four other miRNAs to detect early breast cancer [4]. However, the biological functions and potential molecular mechanisms of miR-6875 in HCC remain unexplored. In our study, miR-6875-3p was found to be highly expressed in HCC tissues and cell lines, and positively correlated with the degree of malignancy and staging of tumors. Based on in vitro and in vivo experiments, it was determined that down-regulation of miR-6875-3p expression inhibited tumor proliferation, invasion and metastasis. Furthermore, we also found that the OS and RFS rates of HCC patients in the low miR-6875-3p expression group were significantly higher than those in the high expression group. These results suggested that miR-6875-3p may act as a cancer-promoting factor in HCC, and abnormally elevated miR-6875-3p may be a predictor of poor prognosis in HCC patients.

miRNAs perform their functions through inhibiting the expression of target mRNAs. Our study confirmed that the tumor promoter role of miR-6875-3p in HCC, but the underlying mechanism remains unclear. Therefore, we speculated that BTG2 may be a candidate target gene of miR-6875-3p using TargetScan, PicTar and miRanda databases. Then, the luciferase reporter assay results indicated that miR-6875-3p regulated BTG2 by directly acting on its 3’-UTR. To further verify whether the effect of miR-6875-3p on HCC cells was achieved by directly inhibiting the BTG2 expression, we down-regulated the expression of BTG2 in miR-6875-3p-silencing cells and detected the cell proliferation and metastasis. The results showed that the proliferation and metastasis of tumor cells were significantly increased, indicating that BTG2 is a mediator in which miR-6875-3p functions.

Studies have shown that BTG2 plays a crucial role in tumor occurrence and development as a tumor suppressor gene [13]. BTG2 expression is decreased in various tumor tissues such as breast cancer, lung cancer, bladder cancer and HCC [18–21], and the low expression of BTG2 is associated with the aggressive clinical manifestations and poor prognosis [10, 11, 22]. The results of our study were consistent with these previous reports.

The molecular mechanism of miR-6875-3p-BTG2 axis promoting the proliferation, invasion and metastasis of HCC cells remains unclear. The results of microarray analysis suggested that the FAK pathway is the primary signal pathway with the changes of BTG2 expression in HCC cells. FAK, as a protein kinase, can promote tumor progression and metastasis, and its high expression is closely related to several progressive solid tumors [23]. It has been reported that FAK/Akt signaling pathway is correlated with the invasion and metastasis of various tumors including HCC [24–27]. Lim et al. [28] discovered that BTG2 can inhibit tumor invasion and metastasis by inhibiting the activation of FAK pathway. In the present study, down-regulation of miR-6875-3p expression reduced the phosphorylation levels of FAK and Akt, whereas silencing BTG2 abolished the inhibitory effect of low-expressed miR-6875-3p on FAK/Akt phosphorylation. All three databases suggested that miR-6875-3p/BTG2 can promote FAK/Akt phosphorylation. Furthermore, PF573228 can counteract the promotion of proliferation, invasion and metastasis by miR-6875-3p, indicating that miR-6875-3p induces the proliferation and metastasis of HCC through the FAK/Akt signaling pathway. A recent study revealed that BTG2 inhibited the activation of FAK pathway by down-regulating reactive oxygen species (ROS) in tumor cell mitochondria [26], but the specific mechanism of miR-6875-3p-BTG2 axis regulating FAK/Akt pathway in HCC cells remains unclear and needs to be further explored.

EMT and stem cell-like properties are critical for the distant metastasis of HCC cells and the formation of new tumors [29, 30]. Our studies have shown that miR-6875-3p can regulate EMT, enhance the stem cell-like characteristics, and promote invasion and metastasis of HCC cells in vitro and in vivo; while down-regulation of miR-6875-3p has the opposite effect. Wang [14] et al. also reported that the FAK/PI3K/AKT signaling pathway was involved in the regulation of EMT in HCC cells. Therefore, we concluded that miR-6875-3p may also regulate EMT of HCC via the FAK/Akt pathway. However, the specific mechanism of miR-6875-3p regulating EMT and stem cell-like characteristics of HCC cells still needs to be further explored.

In summary, we have first demonstrated the biological functions of miR-6875-3p in HCC in vitro and in vivo. We found that miR-6875-3p enhances EMT and stem cell-like properties, and promotes the proliferation, invasion, and metastasis of HCC cells via the BTG2/FAK/Akt pathway. Therefore, this study may provide new predictive indicators and therapeutic targets for HCC treatment strategies.

## Conclusions

In the current study, our study demonstrates that miR-6875-3p is highly expressed in HCC tissue and cells, and its elevated expression is positively correlated with tumor staging, size, degree of differentiation, vascular invasion and poor prognosis in HCC patients. In addition, our study confirms that miR-6875-3p regulates EMT, enhances stem-like cell characteristics, and promotes the proliferation, invasion and metastasis of HCC cells. Furthermore, this study is the first report demonstrating that miR-6875-3p functions by regulating the BTG2/FAK/Akt signaling pathway to promote HCC cells proliferation, invasion and metastasis. Thus, our findings suggest that miR-6875-3p may be a new predictive indicator of survival and prognosis in patients with HCC and may serve as a target for the diagnosis and treatment of HCC.

## Additional files

Additional file 1: **Figure S1.** Microarray analysis showed that the expression of miR-6875-3p was significantly increased in HCC tissues compared with para-carcinoma tissues. (JPG 402 kb)

Additional file 2: **Figure S2.** The relationship of miR-6875-3p and EMT markers expression in HCC tissues. A, B Expression of miR-6875-3p was inversely correlated with the mRNA level of the epithelial markers (E-cadherin and а-catenin) via qRT-PCR. C, D Expression of miR-6875-3p was correlated with the mRNA level of mesenchymal markers (N-cadherin and Vimentin) via qRT-PCR. (Spearman’s correlation analysis). (JPG 382 kb)

## Abbreviations

3’-UTR: 3’-untranslated region
EMT: Epithelia-mesenchymal transition
HCC: Hepatocellular carcinoma
ISH: In situ hybridization
ROS: Reactive oxygen species
