# Supplementary figures and images for: MiR-6875-3p promotes the proliferation, invasion and metastasis of hepatocellular carcinoma via BTG2/FAK/Akt pathway
Source: J Exp Clin Cancer Res. 2019 Jan 8;38:7. doi: 10.1186/s13046-018-1020-z (PMC6323674; doi:10.1186/s13046-018-1020-z)

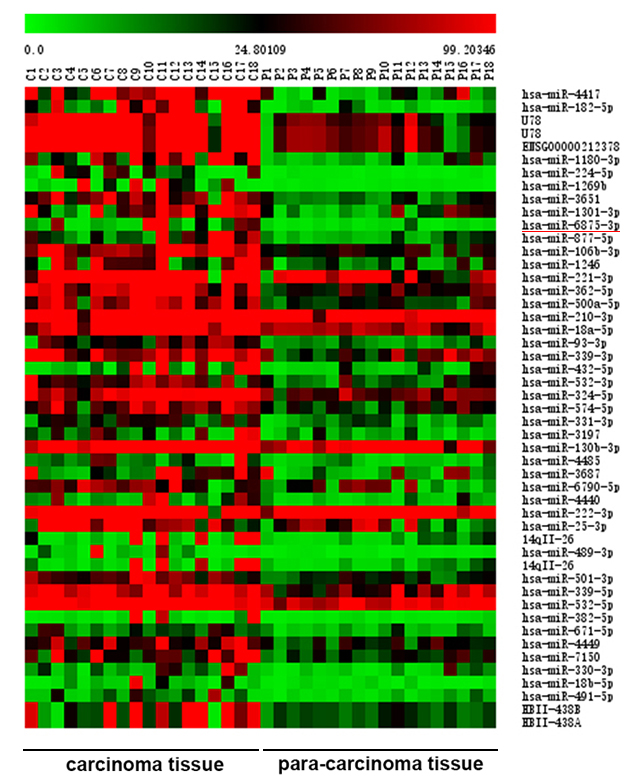

Supplement: Supplementary file 1 — Figure S1. Microarray analysis showed that the expression of miR-6875-3p was significantly increased in HCC tissues compared with para-carcinoma tissues. (JPG 402 kb) [file 13046_2018_1020_MOESM1_ESM.jpg]

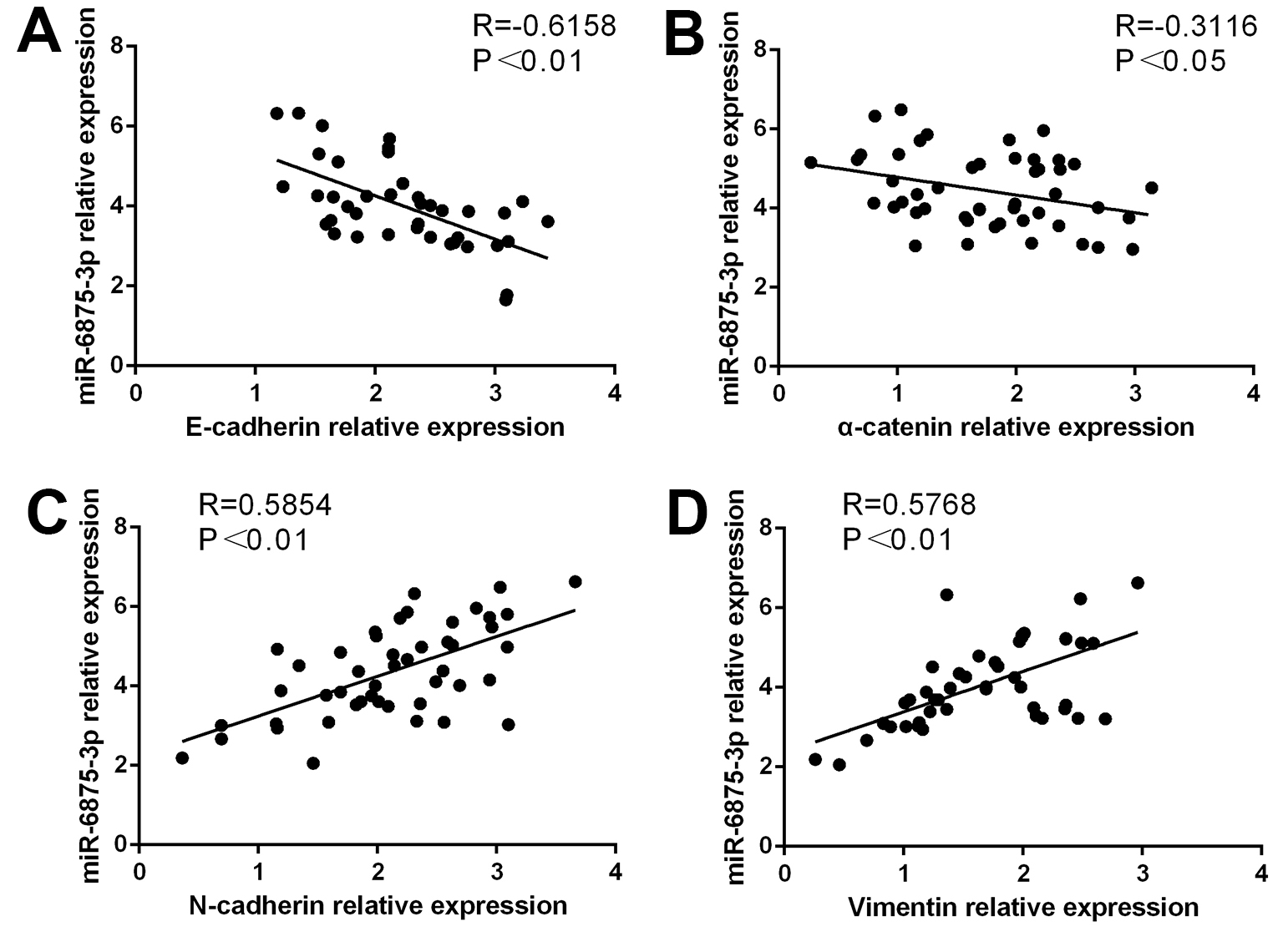

Supplement: Supplementary file 2 — Figure S2. The relationship of miR-6875-3p and EMT markers expression in HCC tissues. A, B Expression of miR-6875-3p was inversely correlated with the mRNA level of the epithelial markers (E-cadherin and а-catenin) via qRT-PCR. C, D Expression of miR-6875-3p was correlated with the mRNA level of mesenchymal markers (N-cadherin and Vimentin) via qRT-PCR. (Spearman’s correlation analysis). (JPG 382 kb) [file 13046_2018_1020_MOESM2_ESM.jpg]
